# Tripal v1.1: a standards-based toolkit for construction of online genetic and genomic databases

**DOI:** 10.1093/database/bat075

**Published:** 2013-10-25

**Authors:** Lacey-Anne Sanderson, Stephen P. Ficklin, Chun-Huai Cheng, Sook Jung, Frank A. Feltus, Kirstin E. Bett, Dorrie Main

**Affiliations:** ^1^Department of Plant Sciences, University of Saskatchewan. Saskatoon, SK Canada, ^2^Department of Horticulture, Washington State University. Pullman, WA, USA and ^3^Department of Genetics and Biochemistry, Clemson University. Clemson, SC, USA

## Abstract

Tripal is an open-source freely available toolkit for construction of online genomic and genetic databases. It aims to facilitate development of community-driven biological websites by integrating the GMOD Chado database schema with Drupal, a popular website creation and content management software. Tripal provides a suite of tools for interaction with a Chado database and display of content therein. The tools are designed to be generic to support the various ways in which data may be stored in Chado. Previous releases of Tripal have supported organisms, genomic libraries, biological stocks, stock collections and genomic features, their alignments and annotations. Also, Tripal and its extension modules provided loaders for commonly used file formats such as FASTA, GFF, OBO, GAF, BLAST XML, KEGG heir files and InterProScan XML. Default generic templates were provided for common views of biological data, which could be customized using an open Application Programming Interface to change the way data are displayed. Here, we report additional tools and functionality that are part of release v1.1 of Tripal. These include (i) a new bulk loader that allows a site curator to import data stored in a custom tab delimited format; (ii) full support of every Chado table for Drupal Views (a powerful tool allowing site developers to construct novel displays and search pages); (iii) new modules including ‘Feature Map’, ‘Genetic’, ‘Publication’, ‘Project’, ‘Contact’ and the ‘Natural Diversity’ modules. Tutorials, mailing lists, download and set-up instructions, extension modules and other documentation can be found at the Tripal website located at http://tripal.info.

Database URL: http://tripal.info/

## Introduction

The human genome project, one of the largest genome sequencing projects undertaken, began in 1990 at an initial cost estimate of $3 billion. The project took 13 years to complete by a large international group of collaborators and produced a high-quality genome assembly (1). Similarly, genomes for other model species were sequenced and online interactive databases were created to allow exploration of the genome sequences, genetic data and annotations for the species (2–7). Improvements in sequencing and other high-throughput data collection technologies have dramatically decreased the cost, personnel and time requirements for large-scale genomic and genetic projects. These improvements have application to evolutionary biology, population genetics and complex trait analysis as well as application in breeding programs. Moreover, massive numbers of polymorphisms identified between individuals can be used to develop better genetic markers with potential for associations with complex traits (e.g. genome-wide association studies and genome selection).

Online resources for model organisms were historically provided by dedicated groups of individuals with programming and Information Technology systems expertise who worked closely with researchers. Despite the decrease in cost, the time and personnel required to perform large-scale genomic and genetic studies, the construction of online databases for visualization and data mining has become more challenging for research groups because of the volume and complexity of these large-scale data sets. As the genome sequences and ancillary analyses of non-model species are increasingly made available, a common platform for data storage, visualization and the ability to integrate with other online tools and functionality is important. A common database platform encourages use of open standards, such as controlled vocabularies and open data formats, and improves the capacity for data exchange. Sites that use a similar infrastructure find it easier to share data and collaborate on development of new tools.

The goal of Tripal is to provide a high-quality, easy-to-administer, intuitive, modular database infrastructure that affords unique customizations for any specific research community. Tripal is an open-source, freely available, standards-based toolkit for storage, integration and dissemination of biological data. A key role of Tripal is to reduce the time and costs for deployment of data-rich genome databases. Tripal uses the GMOD Chado database schema (8, 9) for storage of biological data. Chado relies heavily on community-developed controlled vocabularies [such as the Sequence and Gene Ontologies (10, 11)], supports a broad set of biological data classes and is developed and supported by an active community. Tripal integrates Chado with Drupal, a popular content management system (http://www.drupal.org), and has been used to construct genomic databases with resources for breeders such as Knowpulse: Pulse Crop Genomics & Breeding (http://knowpulse2.usask.ca/portal) (12, 13), and for genome databases such as the Genome Database for Rosaceae (http://www.rosaceae.org) (14), the Banana Genome Hub (http://banana-genome.cirad.fr/) (15), CottonGen (http://www.cottongen.org/) (16), Cacao Genome Database (http://www.cacaogenomedb.org), Hardwood Genomics (http://www.hardwoodgenomics.org/), the Fagaceae Genome Web (http://www.fagaceae.org) (17), the Citrus Genome Database (http://www.citrusgenomedb.org) (18), the Marine Genomics Database (http://www.marinegenomics.org/) (19), the WA Cereals Database (http://cereals.bioinfo.wsu.edu), the Vaccinium Genome Database (www.vaccinium.org) the Cool Season Food Legume Database [www.coolseasonfoodlegume.org] and others. Tripal has also been used for construction of network biology databases such as the GeneNet Engine (http://sysbio.genome.clemson.edu/) for cereal grasses (20).

Tripal uses Chado as its underlying database schema. Chado is an open-source freely available schema with a set of database tables dedicated to storage of biological data, with specific attention to genomic and genetic data. Chado is supported by several software packages either directly or indirectly, including: GBrowse, a popular genome browser, which can read directly from a Chado database (21); Maker, a genome annotation pipeline that can export results to Chado (22); Apollo, a genome annotation curation tool that can read directly from a Chado database (23); Ergatis, a workflow construction toolkit for bioinformatics analysis that can load data into Chado (24); Chado can be exported for use with BioMart, a federated database allowing data mining from disparate aggregating sources (25, 26).

Drupal is a popular content management system that services both small and enterprise-level websites and allows for construction and maintenance of web pages by non-computer programmers. User management functionality allows privileged users to access private data and add or edit content. Drupal also provides an Application Programming Interface (API) that enables computer programmers to create new functionality. Using this API, thousands of user-contributed extensions, or modules, have been created for Drupal by third-party developers to extend the functionality. Those extensions are freely shared on the Drupal website. A site developer can download an extension to add functionality to the site. Tripal is, therefore, a suite of extension modules for Drupal constructed using the Drupal API and provides an interface to the Chado database.

Other commonly used tools can be integrated into a Tripal-based site. For example, the CottonGen site (http://www.cottongen.org) integrates the genome viewer, GBrowse (21), the metabolic pathway viewer, PathwayTools (27), as well as other scientific tools. The use of Drupal extensions and customizations made via the Drupal API on the CottonGen site enabled construction of a conference registration and abstract submission interface for the International Cotton Genome Initiative Conference held in Raleigh, NC, USA, in October of 2012. This conference interface is present along-side genomic and genetic data made available via Tripal and demonstrates the power of Drupal and Tripal to construct a community research database such as the CottonGen website.

Tripal was originally released in 2009 by the Clemson University Genomics Institute with minimal support for Chado. Through additional collaboration with groups at Washington State University, and the University of Saskatchewan, a more robust version was released in 2011 and formally introduced by Ficklin *et al.* (28). Herein, we describe recent improvements to the Tripal package as part of version 1.1. For this article, a demonstration site has been created and is available at http://tripal.gmod.oicr.on.ca/1.1. All screenshots herein have been taken from this site. Additionally, tutorials, documentation, a developer’s handbook, the Tripal API specifications and mailing lists are all available at the Tripal website (http://tripal.info).

## Tripal software

**Figure 1.**
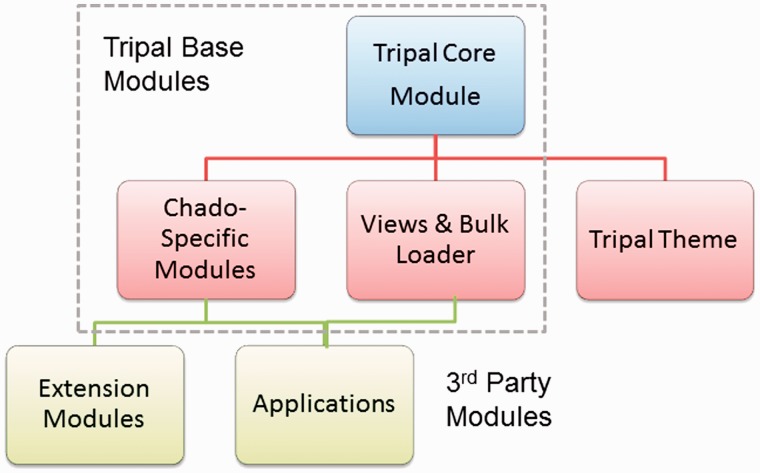
Tripal modules are organized in a hierarchical structure. The Tripal Core module, Chado-specific modules, the Tripal Views and Bulk Loader modules comprise the base Tripal package. Extension modules and applications build on the Tripal API provided by the base modules, but are available separately. The Tripal theme is a separate entity, but is dependent on the Tripal Core module. The Tripal Theme provides all templates for the base modules.

Tripal is a set of Drupal modules that are organized hierarchically. The ‘Tripal Core’ module, Chado-specific modules and the ‘Tripal Views’ and ‘Bulk Loader’ modules all form the base Tripal package. The Tripal theme and extension modules are available separately to extend the functionality of a Tripal site (Figure 1). Historically, Tripal has provided functionality for display of organisms, genomic features (e.g. genes, mRNA, contigs, ESTs, etc.), stocks (germplasm, either natural or mutant, can be stored as stocks), genomic libraries, computational analyses and limited integration of Chado tables for Drupal Views. It also provided loaders for files in FASTA, GFF and Open Biomedical Ontology (OBO) formats. Extension modules were available for loading results from analyses such as BLAST (29), InterProScan (30) and KEGG/KAAS (31, 32), and tools such as Blast2GO (33). Tripal also included a jobs management system for launching long-running jobs (such as data loading), as well as, a mechanism for creating materialized views. Additionally, a set of simple PHP template files, referred hereafter as ‘default templates’, facilitated customization of the display of data pages containing data from the Chado database. Descriptions of these features from previous versions of Tripal, as well as screen shots can be found in the publication by Ficklin *et al.*(28).

More recently, Tripal release v1.0 (March 2013) supported the Chado version 1.2, added improvements in performance for the GFF, FASTA and OBO loaders, used database transactions for data loading, added new modules supporting Maps (genetic and genomic) and the new natural diversity tables of Chado v1.2 (9). It also introduced the ‘Bulk Loader’ module and fully integrated all Chado tables with Drupal Views. Site administrators could also add custom tables to Chado as needed using a web interface. Tripal v1.1 (June 2013) includes improvements to existing code and introduces a new Publication module that queries PubMed (http://www.ncbi.nlm.nih.gov/pubmed) and the USDA National Agricultural Library (NAL; http://agricola.nal.usda.gov/) for importing relevant publications into Chado. Additions and improvements to the Tripal API are also included. The Tripal API provides programmatic access to data in the Chado tables and affords the ability for other programmers to create new modules that extend the functionality of Tripal. The following sections provide descriptions of the new Chado-specific modules, the ‘Bulk Loader’ module, the ‘Tripal Views’ module and the custom tables interface.

## New Chado-specific modules

### The Project module

**Figure 2.**
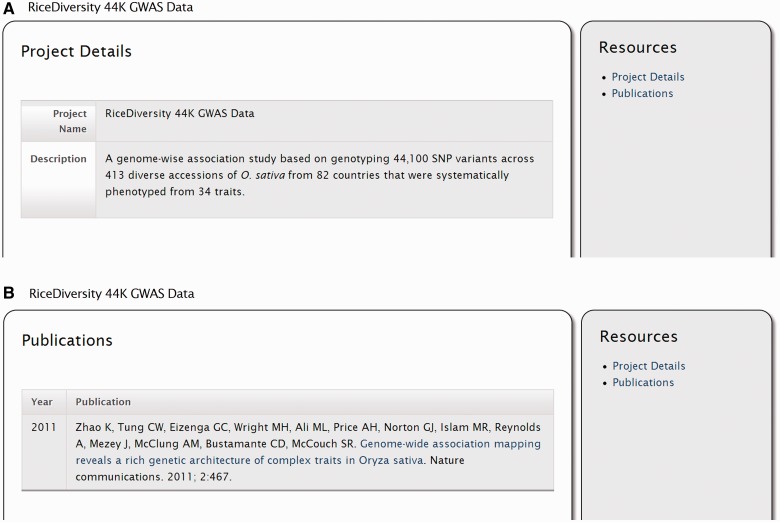
Screenshots of the project page. (**A**) The project details page provide the project name and a description. (**B**) Publications associated with the project. This information is available by clicking the ‘Publications’ link in the right sidebar.

The ‘Project’ module displays pages for projects. ‘Projects’ are important for the new natural diversity tables in that experiments stored in the natural diversity tables can be grouped by projects. An example project can be seen in Figure 2. The information shown is for a project corresponding to a Genome-Wide Association Study (GWAS) for *Oryza sativa* (rice) that was recently performed (34). Sample data from this GWAS has been loaded into the demonstration site to provide example screenshots.

### The Contact module

**Figure 3.**
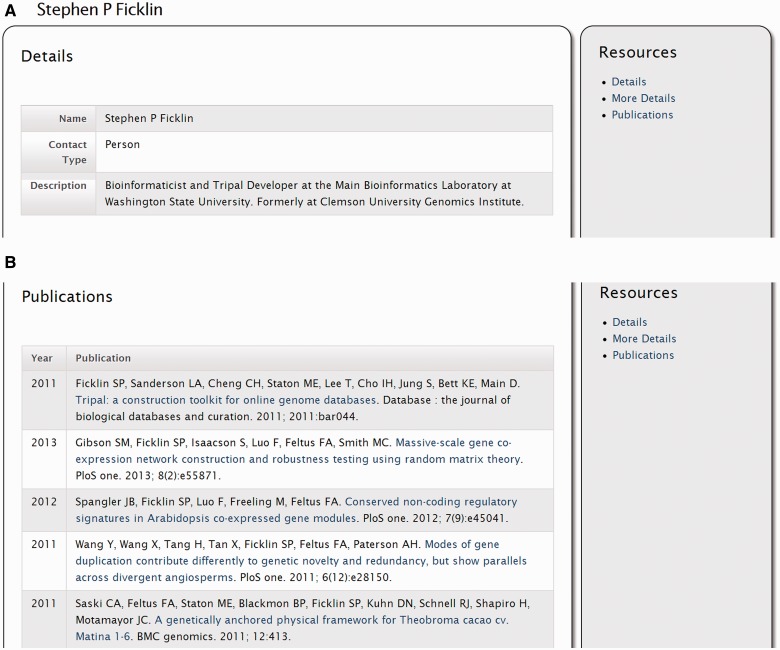
Screenshots of the contact page. (**A**) The contact details provide the name, contact type and a brief description. Additional details are available by clicking the ‘More Details’ link on the right sidebar (not shown). (**B**) If the contact is associated with publications those are shown by clicking the ‘Publications’ link on the right sidebar.

The ‘Contact’ module provides pages for viewing information about people or organizations that are relevant to the data contained in the site. Figure 3 provides a screen shot of an example contact page for an individual. A privileged user can create a new contact using Drupal’s content creation interface, and can select the type of contact (e.g. a person, or organization) and assign properties such as the affiliation, address, institution, lab and more. If the individual is an author on a publication and the author is linked to the publications in the underlying Chado database, then a list of that contact’s publications will be provided (Figure 3b). Additionally, any relationships between contacts (such as membership within an organization) will also be displayed if present in the database.

### The Feature Map module

**Figure 4.**
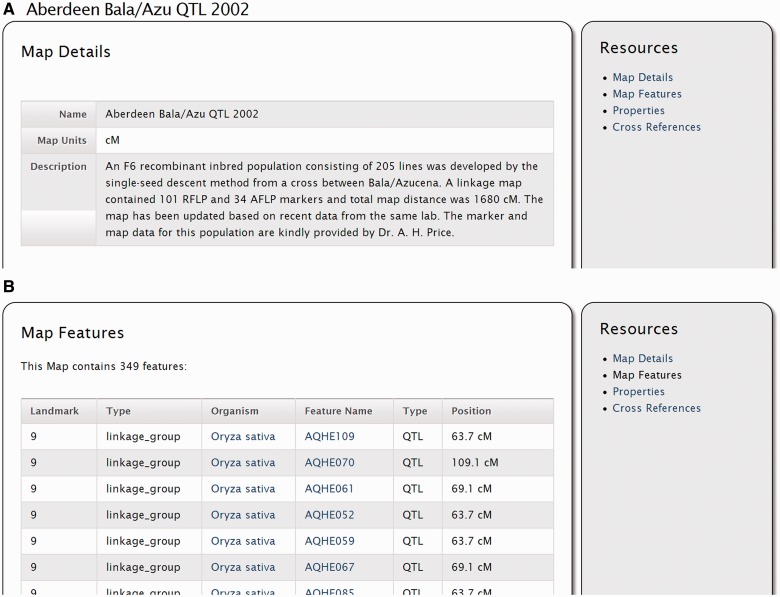
Screenshots of a genetic map page. (**A**) The map details provide the name of the map, the map units and a brief description. Additional details are available by clicking the ‘More Details’ link on the right sidebar (not shown). (**B**) If features (e.g. genetic markers) are associated with a map, they are listed by clicking on the ‘Properties’ link of the right sidebar.

The ‘Feature Map’ module is intended to display information about maps, such as genetic, physical or other maps. Typically, a map will be associated with a set of landmark features, such as linkage groups or chromosomes, and a set of oriented features, such as genetic markers. The Map page, shown in Figure 4, shows information about a genetic map borrowed from the Gramene (4) database for *O. sativa* (rice). Clicking on the ‘Map Features’ link on the ‘Resources’ sidebar will provide a list of features and the landmark on which they are localized. In this case, a list of Quantitative Trait Loci (QTLs) in that genetic map is shown. Additionally, a cross-reference to Gramene is provided with a link to the genetic map viewer at Gramene, and any properties associated with the map.

A privileged user can manually create maps and add relevant properties by using Drupal’s content creation interface. When large numbers of maps or localized features to those maps must be loaded, the ‘Bulk Loader’ (described later) is the ideal mechanism for loading the data. Briefly, the privileged user would prepare a tab-delimited file containing the maps and its localized features, create a bulk loading template and then load the file.

### The Genetic module

There are two methods for storing genetic information, such as genotypes, in Chado. Originally, a privileged user would add genetic or genomic features, stocks (such as germplasm) and their respective genotypes in the feature, stock and genotype tables. The natural diversity tables of Chado allow for storing metadata associated with the experiment itself such as project name and experimental condition. However, the new Tripal ‘Genetic’ Module handles display of genetic data when the natural diversity tables are not used. The module simply adds links to the resource sidebar of the stock and feature pages if genotype information exists for either data type.

### The Publication module

**Figure 5.**
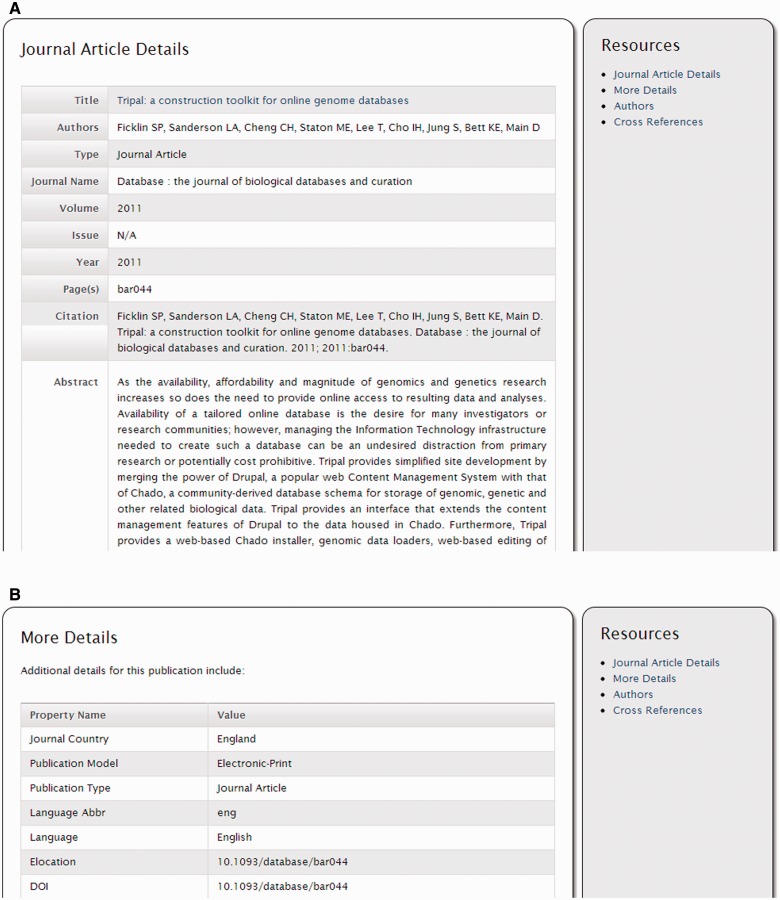
Screenshots of a publication page. (**A**) The publication details provide information about a publication including the title, authors, journal (book or conference, etc.) name, citation and other relevant details. (**B**) Additional details are available by clicking the ‘More Details’ link on the right sidebar.

Chado has database tables for housing information about publications such as journal articles, conference proceedings, books, etc. Additionally, tables exist to link genomic and genetic features, contacts, maps, libraries, projects, stocks and more to publications. The Tripal ‘Publication’ module provides the ability to add publications to the Chado tables and to display those publications. It also automatically shows publications on other pages, such as the feature page, map page, etc. when those data are linked to a publication. Figure 5 shows a screen shot of a publication and its default appearance.

There are two methods for adding publications to the site. A privileged user can add a publication manually or use the publication importer to query NCBI PubMed or the USDA NAL. Because no ontologies in the OBO format contain a broad set of publication types and publication details, a Tripal Publication ontology was created to help classify the properties of a publication. Many of the terms in the Tripal Publication ontology were borrowed from the National Library of Medicine Medical Subject Heading vocabulary.

**Figure 6.**
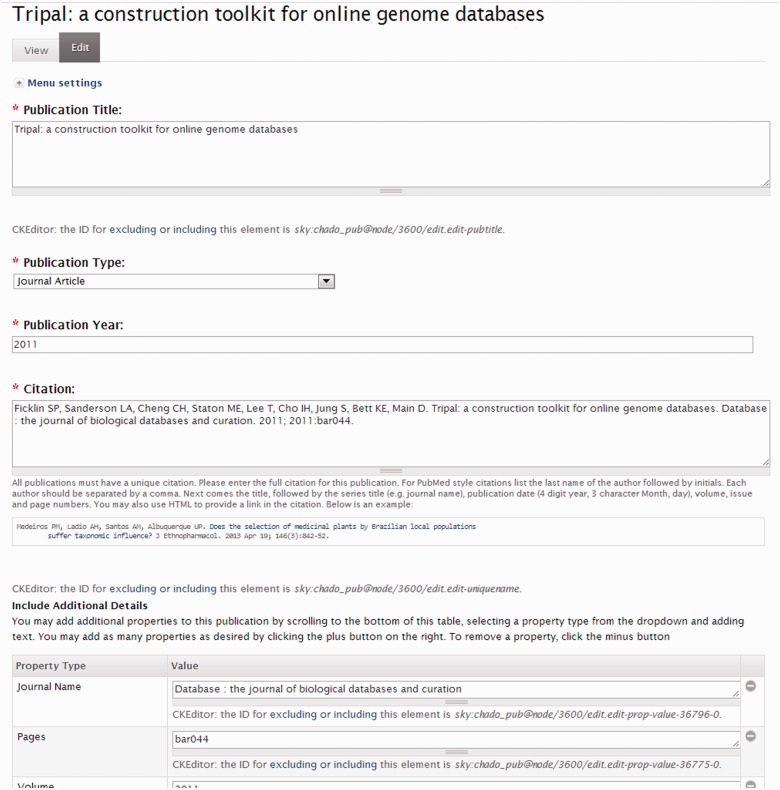
Screenshot of the publication edit page. Here a content manager for the site can edit details about the publication and add any number of properties.

To add a publication manually, a privileged user creates a new publication page using Drupal’s content creation interface and adds details to describe the publication such as the title, year of publication, citation, abstract, publication location, pages, journal issue and volume and more. Figure 6 shows a view of the publication creation page and the interface for adding properties to a publication.

**Figure 7.**
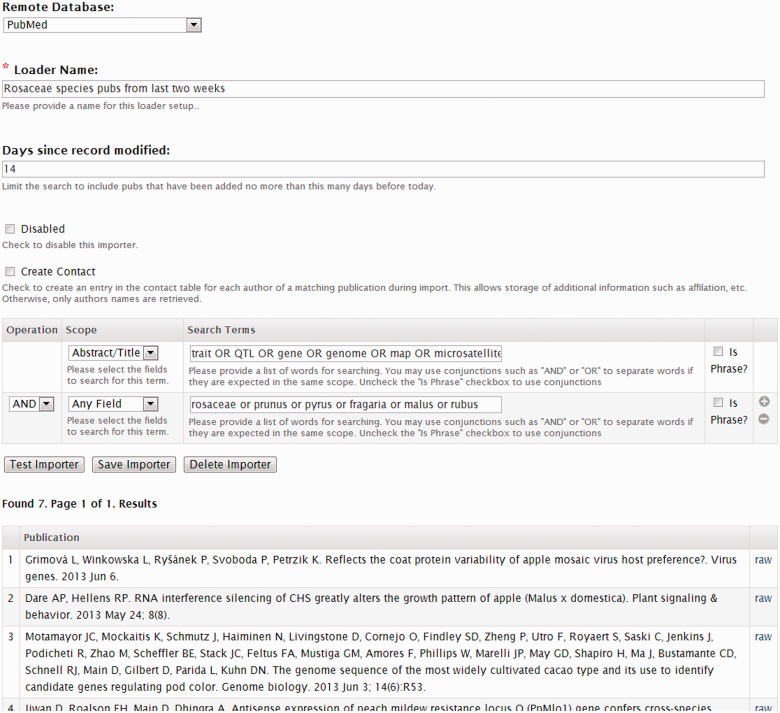
Screenshot of the publication importer. The Tripal publication importer allows a content manager to create a set of search criteria to remotely query a remote publication database such as PubMed. The manager can test the importer query before saving.

Adding publications one at a time can be tedious. Therefore, a privileged user can also add publications in bulk using a publication importer. Figure 7 shows the publication importer interface, which allows the user to specify a set of criteria for querying either PubMed or the NAL databases. The user can save the importer and schedule a periodic execution of the importer. For example, the user can create an importer that looks for new publications for a specific organism within the past 2 weeks and the importer can then be scheduled to run every 2 weeks. This will allow new publications to be automatically added to the database as they become available.

The Publication module also has the ability to create contact records for each author. Using the publication importer interface, the privileged user can enable creation of contact pages for authors. If enabled, new records are added to the contact table of Chado and pages for each author will be created automatically. The Tripal Contact module will then manage display of that information, linking an author to all of his or her publications. However, the publication importer cannot distinguish between different authors with the same name nor recognize the same author whose name is spelled differently between publications.

## The Natural Diversity module

**Figure 8.**
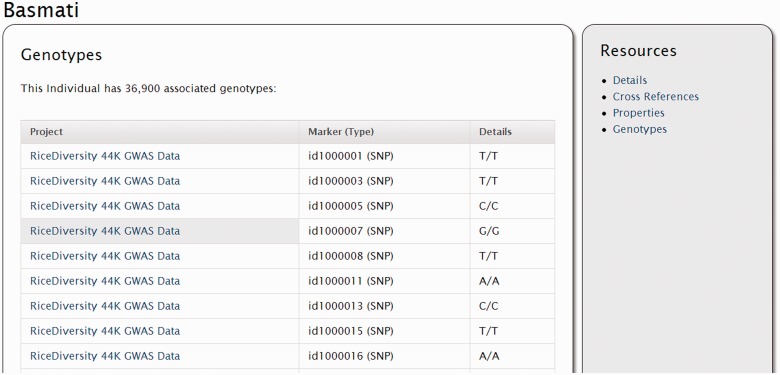
Screenshot of the germplasm (stock) page with associated genotypes. The Natural Diversity Module of Tripal adds genotype information to stocks and features. Here are SNP genotype calls for the germplasm ‘Basmati’.

The ‘Natural Diversity’ module provides a set of templates for display of genotypic, phenotypic or other large-scale assays involving multiple features or stocks (such as germplasm). See the recent publication by Jung *et al.*, 2011 for examples and use cases for the tables of the Chado Natural Diversity module (9). Unlike other modules, there is no content page for this module. However, data stored in these tables are automatically displayed on stock and feature pages. Figure 8 shows a set of genotypes from the rice GWAS study that are associated with the stock (or germplasm accession) named ‘Basmati’. This screenshot displays data stored in natural diversity tables. Phenotypic and genotypic data that is present for any stocks or associated with genetic markers will also appear as a new link on the resources side bar if present.

### Custom tables not in Chado

Chado provides a common database schema for storing biological data and its wider adoption helps promote interoperability of tools and exchange of data between different databases. Chado is an actively developed database schema with updates made periodically, such as the recent addition of the natural diversity tables (9).

**Figure 9.**
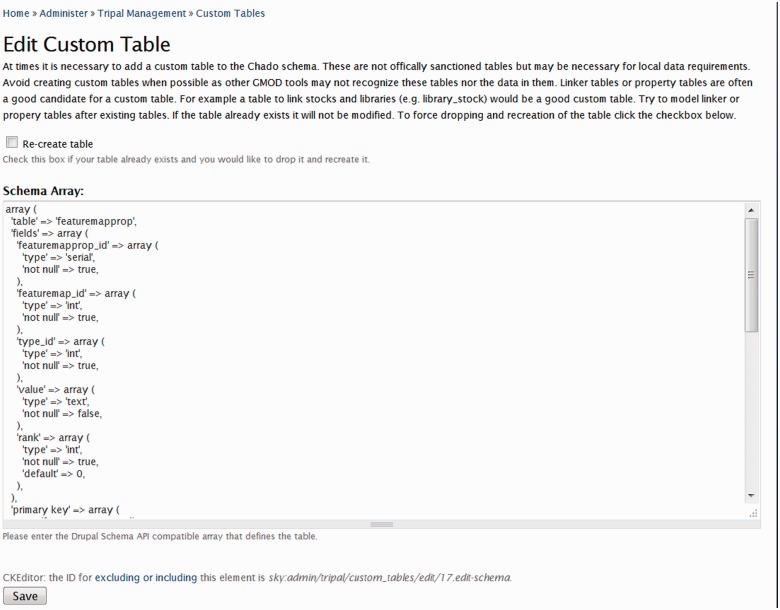
Screenshot of the Custom Table interface. Here a site developer can create custom tables not found in Chado using the Drupal Schema API array structure to define the table.

When adding data to Chado, it is recommended to review the online documentation and best practices to ensure data are stored in similar ways as the broader community. However, there are cases when data cannot be adequately housed in existing tables. Tripal therefore provides a web interface for allowing the addition of custom tables by a site developer. Once a table is added using the Tripal interface, the new custom table immediately becomes available to Tripal’s template files, it can be accessed using the Tripal API functions, can be populated via the ‘Bulk Loader’ and it is made available to Drupal Views (to be discussed later). To add a custom table, the site developer should be familiar with Drupal’s Schema API (https://drupal.org/node/146843), which is used to define the table structure of the custom table. An interface also exists for exporting and importing custom tables. Therefore, a set of custom tables developed for one site can easily be shared with other sites. Figure 9 provides a screenshot of the web interface for creation of a custom table. A corresponding API function also exists for adding custom tables.

It is recommended that custom tables are created only when absolutely necessary. This recommendation is to preserve the interoperability of other tools that support Chado and to preserve the ability for data exchange. In many cases, custom tables should be linker tables and property tables. Linker tables provide foreign key relationships between two Chado tables that do not already have a linker table. For example, there is no table to link features with a contact, or genomic libraries to a stock. Additionally, many tables in Chado have corresponding property tables to store additional properties about the data type. The ‘feature’ table has a ‘featureprop’ table, the ‘stock’ table has a ‘stockprop’ table, but the ‘contact’ table does not have a corresponding ‘contactprop’ table in Chado v1.2. When adding property and linker tables, it is recommended to follow the same naming style and fields used for other linker and property tables in Chado.

In some cases, tables for entire data types may not be present. Such is the case for the tables used by the GeneNet Engine (http://sysbio.genome.clemson.edu), which houses network biology data. To store the data for this site, a suite of tables was created for networks, their nodes, edges and annotations. These custom tables were linked with existing tables of Chado through foreign key constraints. These tables are not part of the Chado v1.2 schema, however, the tables become accessible through the Tripal API and templates.

### The Bulk Loader module

Traditionally, loading of data into Chado via Tripal was limited to a few commonly used file formats such as FASTA, GFF3, GAF, OBO, KEGG/KAAS heir or XML from BLAST and InterProScan. Chado supports many data types that cannot be represented by these file formats, and typically, to populate these other tables, site developers would program custom scripts for data loading. It was desired, therefore, to add a more generic data loader that required no programming and could be sufficiently flexible to load data into any Chado table. The Tripal ‘Bulk Loader’, therefore, was created such that a site developer or curator could create a data template for a tab-delimited file using a web interface, and use that template to load tab-delimited files. Also, many existing data are already stored in flat text files or Excel files. Researchers with data housed in Excel can easily export their spreadsheets to tab-delimited format.

To use the bulk loader, the site developer should have an understanding of the Chado tables, the purpose of each table, the foreign key relationships between tables, the unique constraints and the default values of each table. It is recommended that a site developer consult the documentation and best practices for Chado, or request information from the Chado mailing list before construction of a bulk loader template. This is to be sure that data are stored in similar ways across the Chado user base, which enhances the exchangeability of data. Therefore, a site developer need not have programming experience to load data into Chado. The developer only requires a good understanding of Chado and the bulk loader.

The bulk loader loads tab-delimited files using loading templates (not the same as template files). A loader template provides instructions where each column of the file should be stored in Chado. A bulk loader template is first created using the web interface by the site developer, and then saved for later use. The template can be used to load as many files that match the template as desired.

The bulk loader template consists of a set of records, and each record consists of a set of fields. Records correspond to Chado tables and the fields of a record correspond to fields in the Chado table. The record also contains an operation to perform. For example, the organism table stores information about an organism and requires a genus, species and optionally a common name, abbreviation and a description. If a site developer wanted to load organisms, the tab-delimited file would need to contain at least a genus and species and other optional fields arranged in columns, separated by tab characters. An example data set for Rosaceae species can be seen in Table 1.

**Table 1.** Contents of the example tab-delimited file for loading a set of organisms

Genus	Species	Common name	Abbreviation
Prunus	dulcis	Almond	*P. dulcis*
Fragaria	x ananassa	Strawberry (octoploid)	*F. x ananassa*
Prunus	armeniaca	Apricot	*P. armeniaca*
Malus	x domestica	Apple	*M. x domestica*
Prunus	persica	Peach	*P. persica*
Prunus	serotina	Black Cherry	*P. serotina*
Malus	spp. (apple)	Apple	Malus spp. (apple)
Fragaria	vesca	Woodland strawberry (diploid)	*F. vesca*
Fragaria	spp. (strawberry)	Strawberry	Fragaria spp. (strawberry)
Rosa	sp. (rose)	Rose	R. sp. (rose)
Rubus	sp.	Raspberry	Rubus sp.
Pyrus	spp.	Pear	Pyrus spp. (pear)
Prunus	sp.	Prunus	Prunus sp.
Prunus	spp. (cherry)	Cherry	P. spp. (cherry)

**Figure 10.**
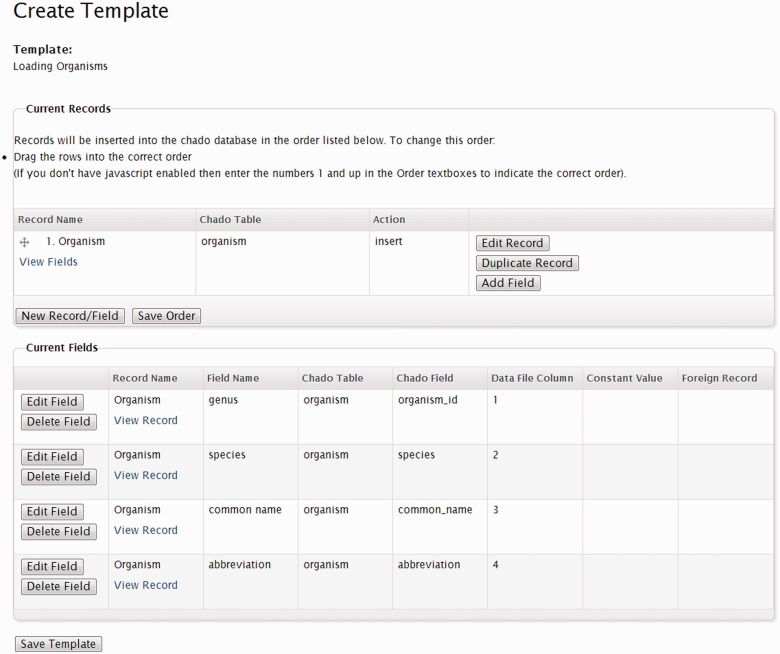
Screenshot of the Bulk Loader interface. This example shows one record with an ‘insert’ operation and four fields: genus, species, common_name and abbreviation. Each field is set to a specific column in the input file. This template can be used for loading organisms into the database.

Using Tripal’s ‘Bulk Loader’ interface, the site developer can load the example organism file by creating a single record for the organism table. The developer would then add four fields to the new record: one for the genus, species, common name, and abbreviation respectively. The developer would associate the genus field with the first column of the tab-delimited file, the species with the second column, etc. The developer then indicates that the operation to perform should be an ‘insert’. Finally, the bulk loader template is saved for later use. Figure 10 provides a screenshot of the bulk loader template for this example organism loader.

When a privileged user desires to load a set of organisms she can simply create a new bulk loader job using the Drupal content creation interface, select the correct loading template and provide the name of the input file. She submits the job and the bulk loader will read the file and populate Chado’s organism table. The next time this template is needed, a new job is created using the same template, and a different input file is specified.

Fields in a record need not be associated directly with columns in the input files, but can contain constant values or refer to the values of previous records in the template. Thus, a field can be one of three types: a data field, a constant field or a referral field. For example, suppose a user needed to load a set of germplasm into Chado’s stock table. The stock table requires a unique name, a type identifier (e.g. germplasm, cultivar, population, etc.) and be associated with an organism. Also suppose that all stocks in any given input file will always belong to the same species.

**Figure 11.**
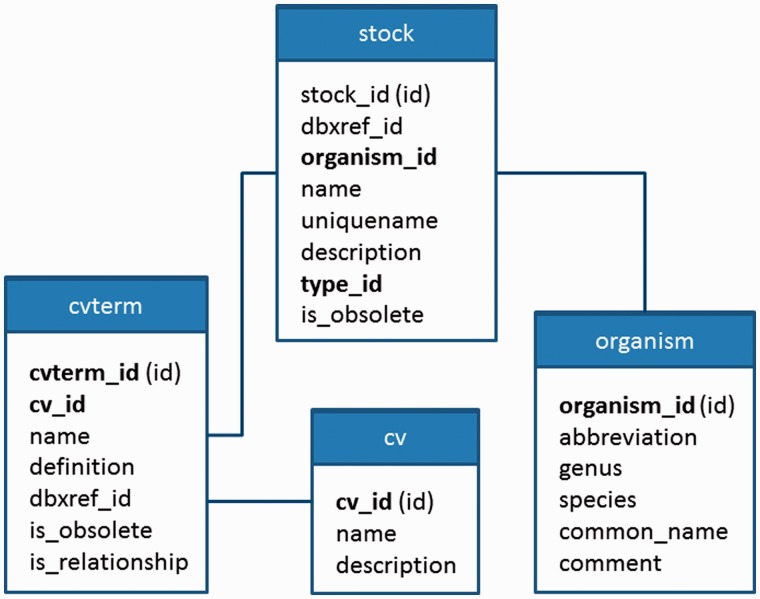
The relationship diagram between stock, cvterm, cv and organism tables. These tables are all needed for storing entries in the stock table. The stock table requires a type, which is stored in the cvterm table. It also requires an organism, which is stored in the organism table. Both are associated to the stock via foreign key constraints with the cvterm_id and organism_id fields, respectively. Fields used in foreign key relationships are bold.

The stock type name (e.g. germplasm) required for each stock is stored in a different table than the ‘stock’ table and the value is associated via a foreign key relationship. The same is true for the organism. Figure 11 shows a relationship diagram of the ‘stock’, ‘organism’, ‘cvterm’ and ‘cv’ tables, all of which will be used for this example. To load a stock, the site developer must therefore add four records to the loading template—one for each of the tables to be queried or inserted.

The first record is for the ‘cv’ table. The ‘cv’ table holds the names of the controlled vocabularies, and because our stock table requires a type, there should be a controlled vocabulary containing stock types. This ‘cv’ record will contain one constant field: the controlled vocabulary name. The vocabulary name should already be present in the database and thus the operation will be a ‘select’, indicating that a database select operation will be performed. Because the vocabulary name will always be the same (our stock type vocabulary is used for all stocks), the site developer will indicate that this field is a ‘constant’ and will manually provide the name of the vocabulary to the field.

Next, the second record will retrieve a record in the ‘cvterm’ table. The ‘cvterm’ table contains the terms of a vocabulary. If all of our stocks are of the stock type ‘germplasm’, then the site developer would create a constant field with an operation to select the term with the name ‘germplasm’. However, the developer wants to ensure that the term is from the correct vocabulary. She does not want to inadvertently retrieve the term ‘germplasm’ from a different vocabulary. Therefore, she creates a new ‘referral’ field for this record. A referral field can be configured to use the values from a previous record. If she associates the new referral field with the previous record for the ‘cv’, she will be indicating that she wants to retrieve a term named ‘germplasm’, but from the stock type vocabulary.

Third, a record selecting the proper organism must be created. Here she must create two fields that will select the proper organism that the stock will belong to. As mentioned previously, she can assume that the germplasm in the input file all belong to the same species. Therefore, she creates two constants fields, one for genus and one for species, but rather than specify the genus and species manually as she did for the stock type vocabulary and the stock germplasm term, she indicates these fields are to be set by the user loading data. In this way, the user can specify the genus and species when they provide the name of the input file.

To this point, all three records have the ‘select’ operation. The fourth and final record will have the ‘insert’ operation. Its fields will consist of a combination of data fields that correspond to fields in the tab-delimited file (for loading the stock unique name, and other optional fields). Referral fields will refer to the ‘organism’ record and the ‘cvterm’ record created previously. This record is responsible for adding each germplasm to the database using the correct type and organism.

The bulk loader also provides more advanced features. Operations are not always simply inserts or selects. Some other operations include the following:

- Select once: prevents the same select from being performed repetitively. It will be performed with the first line of the input file and the values reused for successive lines.
- Continue if no record exists: if when selecting no match is found, the bulk loader will not fail, otherwise it fails and terminates execution.
- Insert once: will only insert the record once on the first line of the input file.
- Select if duplicate: if the record already exists in the database, then select it rather than insert.
- Update if duplicate: if the record matches an existing record in a table via the table’s unique constraint, then update the record.
- Optional: only insert the record if all required fields are present.

Values in tab-delimited files may not have the exact text that should be inserted. Therefore, the data fields may be altered using a regular expression. Regular expressions are a special syntax that helps for matching and replacement of characters in a text string. Many modern programming languages support regular expression and the syntax supported by the Tripal ‘Bulk Loader’ is the same as that documented for PHP. The regular expressions allow the site developer to remove or rewrite unwanted characters from the input values in the text file. Additionally, the bulk loader templates can be exported and imported into other sites. The exported template is simply a PHP array that defines the structure of the loader, and it can be transferred by a simple cut-and-paste action.

## The Tripal Views module

Drupal Views (https://drupal.org/project/views) is an open-source extension module that provides a user interface (UI) for querying the database and creating custom pages. Drupal Views allows site developers to create lists of content retrieved from the Drupal database tables such as users, comments and published pages. By creating filters and exposing the filters to the site user, the listing becomes a simple search form. Alternatively, Drupal Views can be used to construct custom pages for specific data types. Again, no programming is required to build these resources, nor is advanced knowledge of the underlying database structure required. Drupal Views provides extensive flexibility to a site developer for displaying content in novel ways.

The ‘Tripal Views’ module introduced in Tripal v1.0 fully integrates all Chado tables with Drupal Views, thus providing the power of Drupal Views to the Chado tables. Furthermore, Tripal Views describes to Drupal Views all foreign key relationships between Chado tables allowing the site developer to aggregate data into a single list. For example, a list of genetic features could be created that includes the analyses name from which a feature was derived, the feature name, the feature type, the genotypes associated with the feature and with a given biological stock.

By default, Tripal provides several simple search forms preconstructed using Drupal Views. Each instance of custom content created using Drupal Views is referred hereafter as a View. Tripal’s default Views allow a site user to query data in the Chado database. The site administrator can make these simple views available to the site users as needed. When the Tripal Views module is enabled, these views are automatically created and ready for use. A list of simple search tools is provided to the user under a menu item called ‘Search Biological Data’. Because these simple search tools are created using Drupal Views, the site developer can later customize these default Views, or create new ones for more advanced functionality or to handle different data types not yet integrated into the default Tripal templates (e.g. expression or phylogeny data). Each default view is provided by a Chado-specific module. For example, the ‘Feature Module’ provides the feature search view and the ‘Stock Module’ provides the stock search. Therefore, site administrators are only provided with simple search pages from Tripal modules they have enabled.

**Figure 12.**
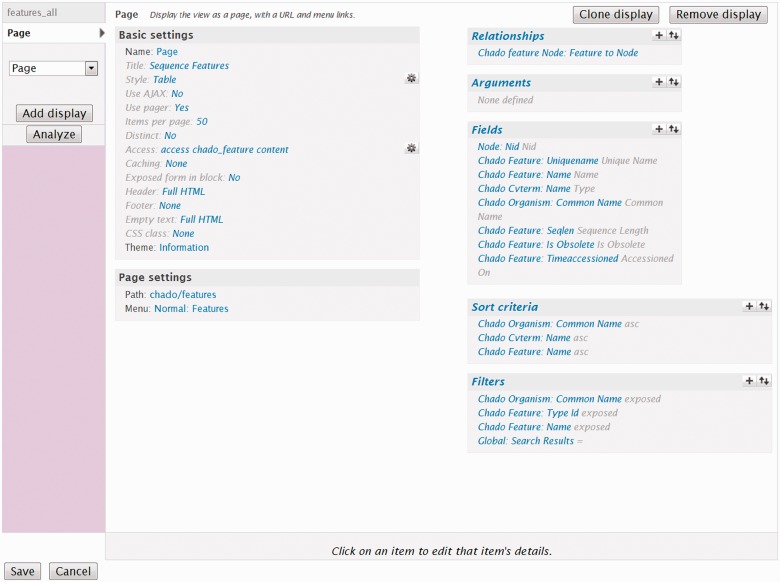
A screenshot of the Drupal Views UI. This screenshot shows the settings for the Feature module default view that allows site users to search for features. The ‘Basic Settings’ section sets some display and behavior criteria for the View. The ‘Fields’ section indicates which fields are to be displayed in the resulting page, and the ‘Sort criteria’ section specifies the order in which features should be displayed. The ‘Filters’ section specifies which features should be displayed. There are three filter criteria: common name, type id and name. While not shown, these fields are exported to the user and appear as form elements when searching (see Figure 13).

**Figure 13.**
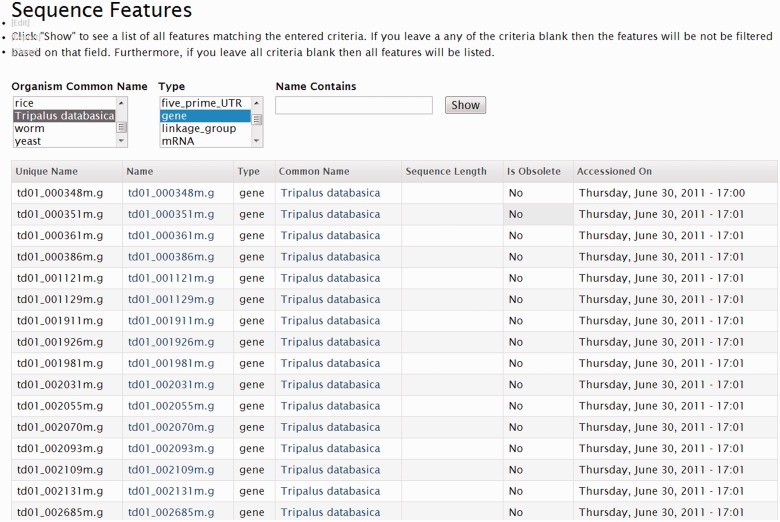
A screenshot of the default feature search View. This search form, with accompanying results is the result of the view described in Figure 12. The form elements for selecting a common name and type are present because they were configured as exposed filters.

Site developers can also customize a Tripal default view by changing the title, enter header or footer text to provide information to their users and restrict which users can see the page. Additionally, the developer can modify display options such as listing results in a table, linked list or grid, indicating how many records are listed at one time and whether a pager for more results is shown. Drupal Views also allows export and import of views from other Drupal sites, allowing site administrators to share their custom views with like-minded communities fostering consistency between community-driven Tripal sites. Figure 12 shows the Drupal Views UI for the default Feature View. This view allows site users to search for features. Figure 13 shows the Feature view when seen by a site visitor. Because the common name and type are exposed filters, the site visitors can set those themselves.

In some cases, the simple search Views provided by Tripal are insufficient for a community. In this case, more advanced views are required. As mentioned previously, the site developer can customize the existing default Views to form a more advanced search tool. Custom tables or materialized views integrated with Drupal Views can be used to construct more advanced search forms, which may be necessary to decrease database query time.

Custom pages for organisms, features, stocks or any other data type can also be created using Drupal Views. These custom pages can be used as an alternative to the default template files. Drupal Views can be useful for creating custom pages when no programming expertise is available to modify the default Tripal template files, although template files will provide the most flexibility for data display. The Drupal Views documentation and tutorials can be consulted to help instruct how to create custom display pages using Drupal Views for Chado content.

For Chado tables to be accessible via Drupal Views, the Tripal Views module integrates them by describing each field of each table. Within Drupal Views special ‘handlers’ are used to indicate how to display (field handler), filter (filter handler) and sort (sort handler) the data. Furthermore, relationships between tables need to be described to Drupal Views. Tripal provides to Drupal Views a set of handlers specific to all Chado tables and indicates all relationships between tables.

**Figure 14.**
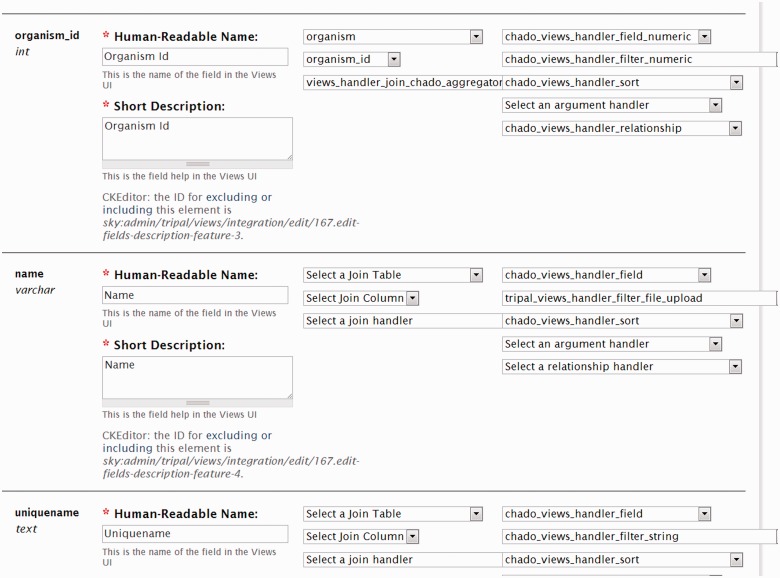
A screenshot of the Views Integration interface. Here a site developer can change the viewable name for a field, the table on which a field can join and set sort, field and filter handlers. Note that for the name field, the filer handler is set to be ‘tripal_views_handler_filter_file_upload’. This handler will provide a form element for uploading feature names when the field is used as a filter and exposed to the site user.

Despite the tables being predefined for Drupal Views, in some cases a developer may want to change handlers. For instance, suppose a site developer wants to create an advanced gene search form, and he/she wants to have a form element that allows the end user to upload a file of gene names to retrieve. Genes are stored in the feature table of Chado, and the default name filter handler only provides a text box where a user can enter the name of the feature to find. The site developer needs a mechanism to change this handler from a text field to a form element that supports file uploads. Tripal does provide a filter handler that includes file upload functionality. Using the Tripal Views Integration infrastructure, the developer can edit the Views integration settings for the ‘feature’ table and set the filter handler for the ‘name’ field to be the file upload handler (as shown in Figure 14). As described previously, custom tables and materialized views are automatically exposed to Drupal Views with default handlers. The site developer may need to edit the Views Integration settings to set any non-default handlers if desired.

Additionally, the Tripal Views Integration page allows a site developer to create alternative settings for any given table. By default, the first instance of settings is given a priority of 10. These are created automatically by Tripal when the Tripal Views module is installed. The site developer can then create a new settings group and set the priority lower. Tripal will provide the lowest priority settings for a table to Drupal Views. Later, the developer has the freedom to switch back to the original settings by simply changing the priorities.

## Alternatives and limitations

At the writing of this manuscript we are not aware of other tools similar to Tripal that integrates a database schema for storage of biological data (i.e. Chado) with a Content Management System (e.g. Drupal) and provides an API to allow for extension by other developers. However, there are other tools that do provide interfaces for Chado. GMODWeb (35) existed before Tripal and provided a Perl framework for construction of online websites. It is still available but no longer developed nor supported by the GMOD project. It does not provide the content management features that Drupal provides. Chado On Rails is another framework (http://gmod.org/wiki/Chado_on_Rails) that provides a Ruby on Rails interface to help developers create web (or other) interfaces into Chado. It can be used to create Chado-based applications (including web applications), but it is not intended to serve solely as a toolkit for website construction and does not provide the loaders, and default pages that Tripal provides. The Drupal Bioinformatics Server Framework (36) provides a set of Drupal modules that can be used to perform data analysis, such as a BLAST search, and can later place results from an analysis into Chado. It is not intended as a web interface for Chado and as such does not provide the generic interface, loading tools, API and other support for Chado that Tripal provides. But, because it is a set of Drupal modules, it can be installed and used along-side of Tripal.

Currently, Tripal is limited because not all the Chado tables are integrated into the default templates. Tripal does provide, however, access to all of the Chado tables via Tripal Views, the Tripal API, and the bulk loader is capable of loading data into any of the existing Chado tables. users who wish to adopt Tripal v1.1 may need to create and update templates to view data in tables that currently are not integrated into the existing templates. An example is the Chado expression module used for storing expression data such as from microarrays. These tables are not yet integrated into the default templates provided by Tripal. The Developer’s Handbook and the API documentation available from the Tripal website can assist a developer extend existing templates for their site.

The Views interface can allow for construction of novel pages and search forms using the web interface without the need for programming. However, the Drupal Views 2 interface is not able to include related data from tables if the foreign key relationships are more than two tables deep. Creation of materialized views [discussed in the Ficklin *et al.* manuscript (28)] can be used to alleviate this problem, but Drupal Views may not be suitable in some cases. Rather, a developer may be required to use the Tripal API to create the desired page or search forms for the site. This limitation has been removed from Drupal Views 3 and, thus, the future work to update Tripal to work with Drupal 7 will also remove this limitation.

Tripal is most easily suited for genomic data. The GFF3, FASTA, OBO and GAF loaders make it relatively easy to import genomic data and the default templates can automatically display these data. The online tutorials demonstrate how to load whole genomic data. Other data are not as easily imported. Currently, the bulk loader is required to import genetic data, genotype, phenotype and other data. Also, the bulk loader is currently limited to loading data housed only in tab-delimited format. In some cases, data already in tab-delimited format may need to be reorganized to ensure existence of values required by Chado’s data integrity constraints. A good understanding of Chado and its unique and foreign key constraints is essential for proper creation of bulk loader templates.

## Future work

There are two major updates to Tripal that are planned for the future. These include an upgrade to ensure compatibility with Drupal 7 and the upcoming release of Drupal 8. Currently, Tripal only supports use on a Drupal 6 website. Drupal 6 is fully supported by the Drupal community but support will cease when Drupal 8 is released (Drupal 7 is already available). Tripal is currently being upgraded to support Drupal 7 and a new release will be made available when that work completes. The Drupal API is not backwards compatible between versions of Drupal. Therefore, upgrading Tripal to support a new version of Drupal (either 7 or 8) requires extensive changes to the code where the Drupal API has changed.

The second major update is to improve the API such that two or more Tripal sites can communicate with one another. The goal is to allow multiple sites to query the other and present results to their users. This reduces the need for users to visit multiple sites to find data of interest and is possible because both use Chado via Tripal.

Additionally, new modules will become available as part of the base package as developed by the Tripal community. There continue to be Chado tables that are not yet integrated into Tripal’s default templates (although, all Chado tables are accessible via the Tripal API, Views and the Bulk loader). Tripal developers are also discussing mechanisms to handle large-scale sequencing, resequencing, expression and phenotying data.
